# U-box E3 ubiquitin ligase PUB17 acts in the nucleus to promote specific immune pathways triggered by *Phytophthora infestans*

**DOI:** 10.1093/jxb/erv128

**Published:** 2015-04-06

**Authors:** Qin He, Hazel McLellan, Petra C. Boevink, Ari Sadanandom, Conghua Xie, Paul R. J. Birch, Zhendong Tian

**Affiliations:** ^1^Key Laboratory of Horticultural Plant Biology, Ministry of Education, Huazhong Agricultural University, Wuhan, Hubei, 430070, China and the National Centre for Vegetable Improvement (Central China), Huazhong Agricultural University, Wuhan, Hubei, 430070, China;; ^2^Division of Plant Sciences, University of Dundee, James Hutton Institute (JHI), Errol Road, Invergowrie, Dundee DD2 5DA, UK; ^3^Dundee Effector Consortium, James Hutton Institute, Errol Road, Invergowrie, Dundee DD2 5DA, UK; ^4^Durham Centre for Crop Improvement Technology School of Biological and Biomedical Sciences, Durham University, Durham DH1 3HP, UK; ^5^Cell and Molecular Sciences, James Hutton Institute, Errol Road, Invergowrie, Dundee DD2 5DA, UK

**Keywords:** Disease resistance, effector-triggered immunity, hypersensitive response, oomycete, plant defence, ubiquitination.

## Abstract

Ubiquitination regulates many processes in plants, including immunity. The E3 ubiquitin ligase PUB17 is a positive regulator of programmed cell death (PCD) triggered by resistance proteins CF4/9 in tomato. Its role in immunity to the potato late blight pathogen, *Phytophthora infestans*, was investigated here. Silencing *StPUB17* in potato by RNAi and *NbPUB17* in *Nicotiana benthamiana* by virus-induced gene silencing (VIGS) each enhanced *P*. *infestans* leaf colonization. PAMP-triggered immunity (PTI) transcriptional responses activated by flg22, and CF4/Avr4-mediated PCD were attenuated by silencing *PUB17*. However, silencing *PUB17* did not compromise PCD triggered by *P*. *infestans* PAMP INF1, or co-expression of R3a/AVR3a, demonstrating that not all PTI- and PCD-associated responses require PUB17. PUB17 localizes to the plant nucleus and especially in the nucleolus. Transient over-expression of a dominant-negative StPUB17^V314I,V316I^ mutant, which retained nucleolar localization, suppressed CF4-mediated cell death and enhanced *P*. *infestans* colonization. Exclusion of the StPUB17^V314I,V316I^ mutant from the nucleus abolished its dominant-negative activity, demonstrating that StPUB17 functions in the nucleus. PUB17 is a positive regulator of immunity to late blight that acts in the nucleus to promote specific PTI and PCD pathways.

## Highlight

Ubiquitin E3 ligase PUB17 functions in the nucleus to regulate transcriptional responses positively in PAMP-triggered immunity and programmed cell death following perception of specific elicitors at the plant cell surface.

## Introduction

Plants are constantly exposed to pathogenic microorganisms such as bacteria, viruses, fungi, and oomycetes. To detect these microbes and to defend against infection, plants have an inducible innate immune system that minimizes the impact of pathogens on plant growth and development (Jones and Dangl, 2006). The first inducible layer of the plant immune system involves detection of conserved, secreted or surface-exposed molecules, called pathogen/microbe-associated molecular patterns (P/MAMPs), leading to the production of reactive oxygen species, activation of mitogen-activated protein kinases (MAPK), deposition of callose in the cell wall, and synthesis of pathogenesis-related (PR) proteins. This is collectively termed pattern-triggered immunity (PTI) (Jones and Dangl, 2006; Dodds and Rathjen, 2010; Thomma *et al.*, 2011). Pathogens secrete effectors that suppress or otherwise manipulate PTI (Block and Alfano, 2011). In turn, effectors can be specifically recognized, directly or indirectly, by nucleotide-binding, leucine-rich repeat (NB-LRR) resistance (R) proteins that trigger a further layer of defence, effector-triggered immunity (ETI) (Jones and Dangl, 2006; Boller and He, 2009). ETI is an accelerated and amplified PTI response, resulting in disease resistance and usually accompanied by a rapid, localized programmed cell death (PCD) known as the hypersensitive response (HR) (Jones and Dangl, 2006).

The disease, late blight, caused by the oomycete pathogen *Phytophthora infestans*, is a significant threat to potato production worldwide (Birch *et al.*, 2012). *P*. *infestans* secretes and delivers pathogenicity proteins into the plant cell cytosol, including potentially hundreds of effectors containing the conserved amino acid motif RXLR (Whisson *et al.*, 2007; Birch *et al.*, 2008; Haas *et al.*, 2009; Cooke *et al.*, 2012). Several RXLR effectors have been shown to be avirulence (AVR) proteins recognized by host cytoplasmic NB-LRR R proteins (Hein *et al.*, 2009; Vleeshouwers *et al.*, 2011). Recent activity has focused on the roles of these RXLR effectors in modifying host processes to the benefit of the pathogen. Amongst them, the effector AVR3a, which is recognized by potato R3a (Armstrong *et al.*, 2005), is able to suppress PCD triggered by the *P*. *infestans* PAMP INF1 (Bos *et al.*, 2006), and a range of other pathogen elicitors (Gilroy *et al.*, 2011), by modifying the activity of the host ubiquitin E3 ligase CMPG1 (Bos *et al.*, 2010). This establishes ubiquitination as a process targeted by *P*. *infestans* to promote the colonization of its hosts (Birch *et al.*, 2009).

During pathogen infection, plant defence responses include remodelling the proteome by protein synthesis (transcription and translation), and post-translational modifications (PTMs) (Stone and Callis, 2007; Kwon *et al.*, 2006). Ubiquitination is a PTM that plays a crucial role during the regulation of plant immune signalling (Lu *et al.*, 2011; Hofmann, 2012; Sadanandom *et al.*, 2012; Stegmann *et al.*, 2012; Duplan and Rivas, 2014). It is initiated by activation of the ubiquitin-activating enzyme (E1) in an ATP-dependent manner, which is then transferred to a ubiquitin-conjugating enzyme (E2). E2 intermediates interact with E3 ligases to catalyse ubiquitin transfer to specific target proteins and/or to auto-ubiquitinate the E3 itself (Yee and Goring, 2009). In this pathway, E3 ligases play a central role in selecting target proteins for ubiquitination and have been shown to be involved in all steps of plant immunity (Dreher and Callis, 2007; Shirsekar *et al.*, 2010; Trujillo and Shirasu, 2010). E3 ubiquitin ligases are classified into four main subfamilies depending on their subunit composition and mechanism of action: HECT, RING, U-box, and cullin-RING ligases (CRLs) (Vierstra, 2009).

A number of PUB (Plant U-Box) E3 ligases are documented as positive or negative regulators of plant immunity (Marino *et al.*, 2012). The E3 ligase SPL11 negatively regulates PAMP-triggered signalling and PTI (Zeng *et al.*, 2004; Li *et al.*, 2012; Liu *et al.*, 2012). In addition, PUB12 and PUB13 are recruited to the FLS2 receptor complex in a BAK1-dependent manner to target FLS2 for degradation (Lu *et al.*, 2011; O’Neill, 2011). Moreover, PUB22 negatively regulates PTI by targeting a subunit of the exocyst complex, Exo70B2, for proteasomal degradation (Trujillo *et al.*, 2008; Stegmann *et al.*, 2012).

In contrast to the negative regulators above, the U-box E3 ubiquitin ligases CMPG1/AtPUB20 (González-Lamothe et al., 2006) and *Arabidopsis* AtPUB17 (Yang *et al.*, 2006), and its functional orthologue in tobacco, NtACRE276 (hereafter referred to as NtPUB17), were demonstrated to act as positive regulators of plant disease resistance. They are required for the HR on perception of the *C. fulvum* Avr9 and AVR4 peptides by the Cf9 and Cf4 receptor-like proteins, respectively, in tomato (Rowland *et al.*, 2005; González-Lamothe et al., 2006; Yang *et al.*, 2006). NtPUB17 and AtPUB17 are functional orthologues as transient expression of AtPUB17 in NtPUB17-silenced lines of Cf-9 tobacco plants can restore the AVR9-triggered HR. Moreover, AtPUB17 is also required for HR mediated by the NB-LRR resistance proteins RPM1 and RPS4 (Yang *et al.*, 2006). Potato PUB17 (StPUB17) was first identified by suppression subtractive hybridization analysis of transcriptional changes after *P*. *infestans* inoculation and it was found to be rapidly up-regulated during infection (Tian *et al.*, 2003). Preliminary work showed that *StPUB17*-RNAi potato plants exhibited enhanced susceptibility to *P*. *infestans* (Ni *et al.*, 2010), suggesting it potentially plays a role in basal immunity to *P*. *infestans*. Currently, it is unknown: (i) what aspects of plant immunity of potential relevance to the host-*P*. *infestans* interaction are positively regulated by PUB17; or (ii) where within the plant cell this E3 ligase acts to promote immune responses.

A deeper investigation is conducted here into the role of StPUB17 in immunity against *P*. *infestans* and the role of PUB17 from solanaceous plants in acting as a positive regulator of PCD. Given that StPUB17 is potentially involved in basal immunity to *P*. *infestans* (Ni *et al.*, 2010), a further hypothesis to be tested was that it contributes to other aspects of pattern-triggered immunity. To that end, it is shown that stable silencing by RNAi of *StPUB17* in potato leads to enhanced *P*. *infestans* colonization, confirming a negative impact of StPUB17 on late blight disease development. A relative of potato in the Solanaceae*, Nicotiana benthamiana,* acts as a host for *P*. *infestans* and thus an alternative system for studying *P*. *infestans*-host interactions (Bos *et al.*, 2010; Saunders *et al.*, 2012; McLellan *et al.*, 2013; King *et al.*, 2014). *N*. *benthamiana* is a model plant for virus-induced gene silencing, cell biology, and transient immune-associated assays, allowing more detailed functional studies to be performed than are feasible in potato. Silencing *NbPUB17* in *N*. *benthamiana* using virus-induced gene silencing (VIGS) also led to enhanced *P*. *infestans* colonization and attenuated early PTI-associated transcriptional responses, indicating that it contributes to more than just cell death pathways. Whereas VIGS of *NbPUB17,* or RNAi silencing of *NtPUB17* in tobacco, each compromised CF4/Avr4 cell death, there was no attenuation of cell death mediated by the *P*. *infestans* PAMP INF1, showing that not all cell-surface perception events leading to PCD require PUB17. In addition, silencing *PUB17* in *N*. *benthamiana,* tobacco or potato had no impact on HR triggered by potato R3a-mediated recognition of AVR3a from *P*. *infestans*. Thus not all *R* gene–mediated PCD requires PUB17. These results implicate PUB17 involvement in the nucleus as a positive regulator of specific defence pathways activated by *P*. *infestans* infection.

## Materials and methods

### Plant materials and microbe strains

*In vitro* potato plantlets were propagated in sterile culture boxes containing MS medium supplemented with 4% sucrose and 0.7% agar and raised in a climate room under controlled conditions (16/8h light/dark cycle at 20 °). Three-week-old plantlets were transplanted and grown in individual pots in a greenhouse at 20–26 ° with humidity above 80%. *Nicotiana benthamiana*, Cf9, and *ACRE276*-RNAi tobacco lines were grown as described in Bos *et al.* (2010) and Yang *et al.* (2006). All *Agrobacterium tumefaciens* cultures were grown at 28 ℃ at 200rpm for 24–48h and spun at 4000 *g*. The pellet was re-suspended in sterile 10mM MES and 10mM MgCl_2_ buffer with 200 µM acetosyringone. The following bacterial optical densities at 600nm (OD600) were used for each assay: 0.1–0.01 for confocal imaging, 0.5 for Western Blot analyses and HR assays, and 0.1 for *P*. *infestans* virulence assays.

### Isolation and sequence analysis of *StPUB17* and *NbPUB17* genes

A full-length *StPUB17* gene was cloned with gene-specific primers from potato cDNA [synthesized from the RNA of E-potato 3 (E3) leaves inoculated with *P*. *infestans* for 36 h]. Primer sequences are shown in Supplementary Table S1 at *JXB* online. The single PCR products were gel purified, then digested with *Eco*RI and *Not*I, ligated into the *Eco*RI–*Not*I-digested pMD18-T vector (Takara, Dalian, China), and transformed into *E*. *coli* strain DH5a for sequencing, resulting in pMD18-T-StPUB17. Full-length NbPUB17 was cloned from *N*. *benthamiana* cDNA with gene-specific primers and then ligated into the pMD18-T vector, resulting in pMD18-T-NbPUB17. Sequence analysis was performed by BLAST (http://blast.ncbi.nlm.nih.gov/). Information on all genes, constructs, and their purpose is included in Supplementary Table S2 at *JXB* online.

### Mutagenesis

The StPUB17^V314I,V316I^ mutant was generated according to the manufacturer’s protocol QuickChange^®^ Site-Directed Mutagenesis Kit (Stratagene) using pDonr201-StPUB17 as a template. The primer sequences used for mutation are shown in Supplementary Table S1 at *JXB* online. Two conserved amino acids of StPUB17, valines at positions 314 and 316, were substituted with isoleucine, resulting in the mutant StPUB17^V314I,V316I^. The mutant StPUB17 was recombined, using LR clonase, into pB7WGF2 or NES-pB7WGF2 for *in planta* assays.

### Cloning of protein fusions

A full-length *StPUB17* gene was cloned from a pMD18-T-StPUB17 plasmid with gene-specific primers modified to contain the Gateway® (Invitrogen) attB recombination sites. Primer sequences are shown in the Supplementary Table S1 at *JXB* online. PCR products were purified and recombined into pDONR201 (Invitrogen) to generate entry clones via BP reactions using Gateway® technology (Invitrogen). N terminal GFP fusions of StPUB17, StPUB17^V314I,V316I^, and NtPUB17 were made by recombining the entry clones with pB7WGF2 using using LR clonase® (Invitrogen). The NES-GFP-StPUB17^V314I,V316I^ was made by recombining the entry clones with NES-pB7WGF2 (the NES signal sequence was inserted with annealed oligonucleotides into the unique *Spe*I site at the beginning of the GFP in the pB7WGF2 vector). They were then transformed into the *Agrobacterium tumefaciens* strain AGL1 virG for *in planta* assays.

### Recombinant protein purification

The fragment of StPUB17 was cloned into the pET28a vector (N terminal HIS-tag), then transformed into the *E*. *coli* strain BL21. The culture was induced by 0.2mM isopropyl-β-d-thiogalactopyranoside (IPTG) at 37 ℃ for about 3h according to the method described by Yang *et al.* (2006). Cells were harvested by centrifugation at 10 000rpm for 20min, and then re-suspended in 8ml of Lysis/Equilibration Buffer (50mM sodium phosphate, 300mM sodium chloride, 5mM imidazole, pH 8 with 0.1% Triton X-100). Then it was sonicated and centrifuged at 10 000rpm for 20–30min to remove genomic DNA, insoluble proteins, and cell debris. The supernatants were incubated and purified according to the protocol of PureProteome Nickel Magnetic Beads (Millipore Corporation). The fused HIS-StPUB17 proteins were eluted with Elution Buffer (50mM sodium phosphate, 300mM sodium, chloride, 300mM imidazole, pH 8) and used for the ubiquitination assays.

### E3 ubiquitin ligase activity assay

The *in vitro* ubiquitination assays were performed according to the Auto-ubiquitinylation Kit (Instruction Manual BML-UW0970; Enzo Life Sciences). Each reaction (50 μl final volume) contained 2.5 μl 20× E1, 2.5 μl 20× E 2, 5 μl 10× Ub E3 ligase buffer, 5 μl 10× ubiquitin, 1 μl 50mM DTT, 2.5 μl Mg-ATP, and 2.5 μl of eluted/bead-bound HIS: StPUB17 proteins or 2.5 μl 20× E3 control (6 μM). Hdm2 RING domain (KW0200) is provided as a positive control of ubiquitin E3 ligase. The reactions were incubated at 37 ℃ for 1h. Quench assays were performed by addition of 50 μl 2× SDS-PAGE gel loading buffer (0.25M TRIS-Cl, pH 6.8, 4% SDS, 10% glycerol, 2% mercaptoethanol, 0.01% bromophenol blue), heated to 95 ℃ for 5min, and analysed by SDS-PAGE electrophoresis. Following blotting, hybridization was performed using ubiquitin antibody (the kit supplied) and Goat Anti-Rabbit IgG-peroxidase antibody (HRP-linked) (Sigma, A0545).

### Western analysis

Leaf discs expressing GFP-StPUB17, GFP-StPUB17^V314I,V316I^, NES-GFP-StPUB17^V314I,V316I^, and GFP-NtPUB17 were harvested at 2 dpi, ground in liquid nitrogen, suspended in 200 μl 2× SDS-PAGE loading buffer, loaded onto a 4–12% TRIS NuPAGE Novex gel, and electrophoresed with 1× MOPS SDS running buffer for 1h at 80V (Invitrogen). All further steps for Western analyses are as described previously by McLellan *et al.* (2013).

### Confocal microscopy

*A*. *tumefaciens* containing GFP-StPUB17 was pressure infiltrated into leaves of 4-week-old wild-type *N*. *benthamiana* plants and the transgenic *N*. *benthamiana* line CB157 (with an mRFP fusion to histone H2B), separately. *A*. *tumefaciens* containing GFP-StPUB17^V314I,V316I^, NES-GFP-StPUB17^V314I,V316I^, and NtPUB17 were pressure infiltrated into leaves of 4-week-old normal *N*. *benthamiana* plants. Cells expressing fluorescent protein fusions were observed using a Leica TCS-SP2 AOBS confocal microscope no more than 2 d post-infiltration, and use a low OD600 (start with 0.01) as described in McLellan *et al.* (2013).

### TRV-based VIGS in *N*. *benthamiana*

Virus-induced gene silencing (VIGS) constructs were made by cloning 348bp and 222bp PCR fragments from *NbPUB17* into pBinary Tobacco Rattle Virus (TRV) vectors (Ratcliff *et al.*, 2001). Primer sequences are shown in Supplementary Table S1 at *JXB* online. A TRV construct expressing GFP, described previously, was used as a control (Gilroy *et al.*, 2007). *A*. *tumefaciens* strains containing a mixture of RNA1 and each NbPUB17 VIGS construct at OD600=0.5 were pressure infiltrated into the two largest leaves of 4-leaf-stage *N*. *benthamiana* plants. Systemic leaves were detached, analysed by qRT-PCR, and used for *P*. *infestans* colonization and HR assays 3 weeks later.

### *Agrobacterium*-mediated transient expression

*Agrobacterium* strains (expressing INF1, R3a/AVR3a, or Cf4/Avr4, Cf4/Avr4/GFP-pB7WGF2, Cf4/Avr4/GFP-StPUB17^V314I,V316I^ Cf4/Avr4/NES-GFP- StPUB17^V314I,V316I^) were infiltrated into leaves of *N*. *benthamiana* wild-type or VIGS plants. Transient expression by agroinfiltration was performed as described previously (Bos *et al.*, 2006; Gilroy *et al.*, 2011). The number of positive HRs (i.e. more than 50% of the inoculated region produces clear cell death) were counted as described previously (Gilroy *et al.*, 2011) and expressed as the mean percentage of total inoculations per plant. The error bars represent ±standard errors (SE) of combined data from at least three biological replicates. One-way ANOVA was performed to determine statistically significant differences.

### Bacterial pathogen strains and pathology tests

Pathogen assays were performed as in Vleeshouwers *et al.* (1999). *P*. *infestans* Ljx18 (race 3.4.7.10.11) and HB09-14-2 race (race 1.2.3.4.5.6.7.8.9.10.11, collected from Hubei Province, China) were used for potato infection after cultured on Rye Agar at 19 ℃ for 2 weeks.

For *N*. *benthamiana* plants and leaves, *P*. *infestans* strain 88069-tdT was inoculated at a concentration of 4×10^4^ sporangia ml^–1^, as previously described by McLellan *et al.* (2013). Sporangia counts with a haemocytometer were performed on 10 dpi leaves from VIGS plants which had been washed with in ddH_2_O to release sporangia and were expressed as sporangia ml^–1^. All the data were analysed by ANOVA.

### Quantitative RT-PCRs

Total RNA from the leaves of potato transgenic lines, *N*. *benthamiana* VIGS plants, and plants that were treated with flg22 for 3h were extracted with TRIzol reagent according to the manufacturer’s recommendations. The cDNA was synthesized and the qRT-PCR conditions are as described previously (McLellan *et al.* 2013). Primers for Real-time PCR are in Supplementary Table S1 at *JXB* online. Gene expression levels were calculated by a comparative Ct method as described by Cikos *et al.* (2007).

### Trypan blue staining

At least three leaves of each line of infected leaves at 5 dpi were collected and stained with trypan blue (0.25mg ml^–1^) solution in a heated water bath, boiling for 1min. Leaves were kept in the staining solution overnight and then de-stained with alcoholic lactophenol solution (95% ethanol:lactophenol=2:1 v/v; lactophenol,phenol:glycerol:lactic acid:water=1:1:1:1 by vol.). Leaves were cleared with ethanol (50%) and stored in glycerol (50%). The representative phenotypes were photographed.

## Results

### StPUB17 functions as an E3 ubiquitin ligase

A transcript (GenBank: EF091878) shown previously to accumulate during *P*. *infestans* colonization of potato by 2 d post-inoculation (dpi) (Ni *et al.*, 2010) encodes a 724 amino acid protein of predicted size 79kDa which is a reciprocal best blast hit (RBBH) of AtPUB17 in *Arabidopsis* and ACRE276 (NtPUB17) in tobacco. Alignment of the corresponding StPUB17 protein with AtPUB17 and NtPUB17 reveals a shared U-box domain (amino acids 300–363 in StPUB17), and three ARM repeat domains (amino acids 430–470, 515–555, 556–594 in StPUB17) in the C-terminal halves of the proteins (see Supplementary Fig. S1 at *JXB* online). To test whether StPUB17 possesses E3 ubiquitin ligase activity, full-length StPUB17 was expressed in *E*. *coli* (BL21) as a HIS-tagged fusion protein and purified by affinity chromatography (see Supplementary Fig. S2A at *JXB* online). In the presence of E1, E2, ubiquitin, and ATP, ubiquitination activity was observed in immunoblots probed with monoclonal antibodies of ubiquitin (see Supplementary Fig. SB at *JXB* online). No ubiquitination was detected in the absence of E1, E2, ubiquitin, or StPUB17, indicating that the latter possesses E3 ligase activity (see Supplementary Fig. S2B at *JXB* online), as demonstrated for AtPUB17 and SlPUB17 previously (Yang *et al.*, 2006).

### *P*. *infestans* colonization is enhanced by RNAi of *StPUB17* in potato and by virus-induced gene silencing of *NbPUB17* in *Nicotiana benthamiana*

**Fig. 1.**
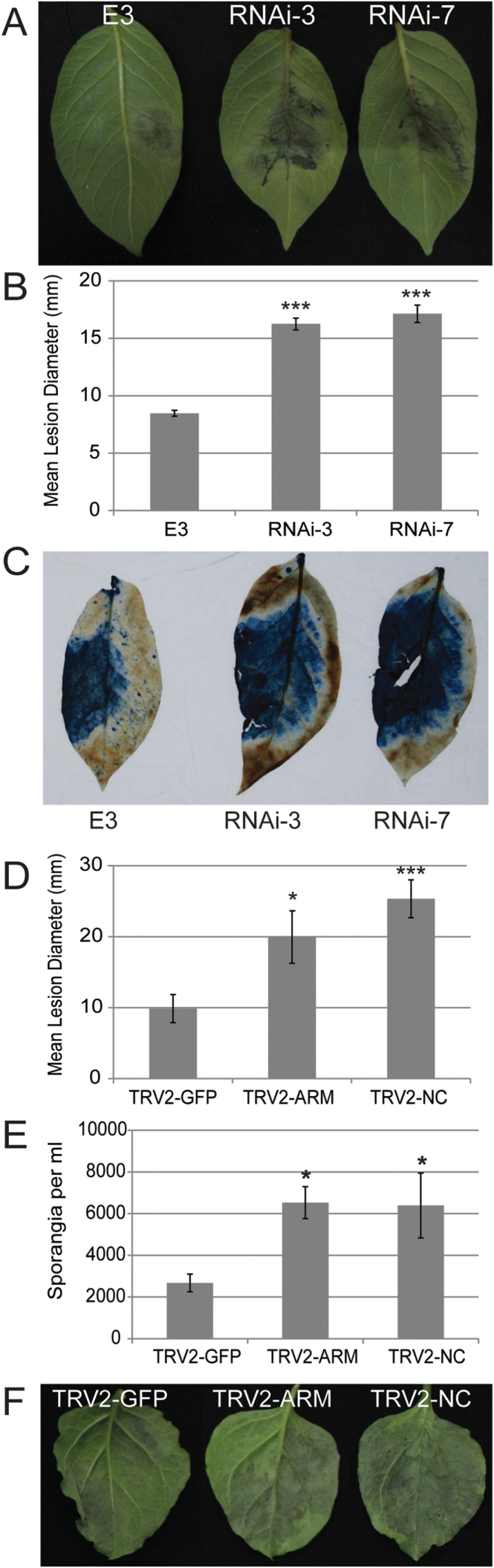
*P*. *infestans* colonization is enhanced by RNAi of *StPUB17* in potato and by virus-induced gene silencing of *NbPUB17* in *N*. *benthamiana*. (A) Representative images of leaves of the Control (E3) and *StPUB17* RNAi potato lines at 4 d post infection (dpi) with *P*. *infestans*. (B) Graph showing mean lesion diameter at 4 dpi with *P*. *infestans* on the Control (E3), and the *StPUB17* RNAi-3 and RNAi-7 potato lines. (C) Trypan blue staining of representative leaves of the Control (E3) and the *StPUB17* RNAi-3 and RNAi-7 potato lines at 4 dpi. (D) Graph showing mean lesion diameter of *P*. *infestans* inoculations at 7 dpi on *N*. *benthamiana* plants silenced for *NbPUB17* compared with the TRV-GFP control. (E) Graph shows the number of sporangia recovered ml^–1^ at 10 dpi from *P*. *infestans*-infected leaves silenced for *NbPUB17* compared with the TRV-GFP control. (F) Representative leaves silenced for *NbPUB17* and TRV-GFP at 10 dpi with *P*. *infestans*. Statistical analysis was carried out using ANOVA with pairwise comparisons performed with a Holm–Sidak test (Holm, 1979). Asterisks denote the *P* value as follows: *P ≤0.05, ***P ≤0.001; error bars show standard error. All experiments are the combination of at least three biological replicates, each using six or seven plants, and inoculations of at least four leaves from each plant.

Previous preliminary results had indicated that RNAi of *StPUB17*, using a portion spanning the U-box-encoding region (see Supplementary Fig. S3A at *JXB* online) in susceptible cultivar E-potato-3 (E3) resulted in enhanced *P*. *infestans* colonization (Ni *et al.*, 2010). As all RNAi lines generated previously had shown similar enhancement of *P*. *infestans* colonization (Ni *et al.*, 2010), just two of these RNAi lines were selected (RNAi-3 and RNAi-7) for further analysis here. Each revealed an approximately 60% reduction in *StPUB17* transcript accumulation (see Supplementary Fig. S3B at *JXB* online). In each line, *P*. *infestans* colonization, measured as lesion diameter, was significantly (*P* <0.001, one way ANOVA) more extensive (Fig. 1A, B), compared with the untransformed cultivar E3. This was strikingly apparent using trypan blue (Fig. 1C), which stains *P*. *infestans* mycelium to reveal the extent of pathogen colonization (Bos *et al.*, 2010).

To investigate the impact of PUB17 on *P*. *infestans–*host interactions further, *StPUB17* was aligned with the corresponding RBBH in the *N*. *benthamiana* genome, designated *NbPUB17*, and two portions were selected, based on them sharing no significant homology with other sequences in potato or *N*. *benthamiana*, to avoid the potential for off-target silencing. Each portion was cloned into the *Tobacco Rattle Virus* (TRV) vector for virus-induced gene silencing.

Constructs TRV::ARM, containing a portion spanning the ARM repeat-encoding region, and TRV::NC, containing a ‘non-conserved’ portion upstream of the U-box (see Supplementary Fig. S4A at *JXB* online), together with TRV::GFP as a control, were inoculated into 3-week-old plants and subsequently challenged with *P*. *infestans* 21 d later. A combination of three experimental replicates, each involving inoculations in quadruple on three leaves on each of six VIGS plants revealed that silencing of *NbPUB17* with either TRV::ARM or TRV::NC resulted in significantly (ANOVA, *P* <0.01) increased *P*. *infestans* colonization compared with TRV::GFP control plants, as measured by lesion diameter, and by number of sporangia recovered from lesions (Fig. 1D–F). QRT-PCR revealed a decrease of *NbPUB17* by 60–80% with either TRV::ARM or TRV::NC constructs, compared with TRV::GFP, in each of the three replicate experiments (see Supplementary Fig. S4B–D at *JXB* online). Taken together, VIGS in *N*. *benthamiana* and RNAi in potato demonstrate that *NbPUB17* and *StPUB17*, respectively, contribute to reducing *P*. *infestans* infection.

### Silencing of *NbPUB17* in *N*.*benthamiana* attenuates early up-regulation of PTI marker genes

**Fig. 2.**
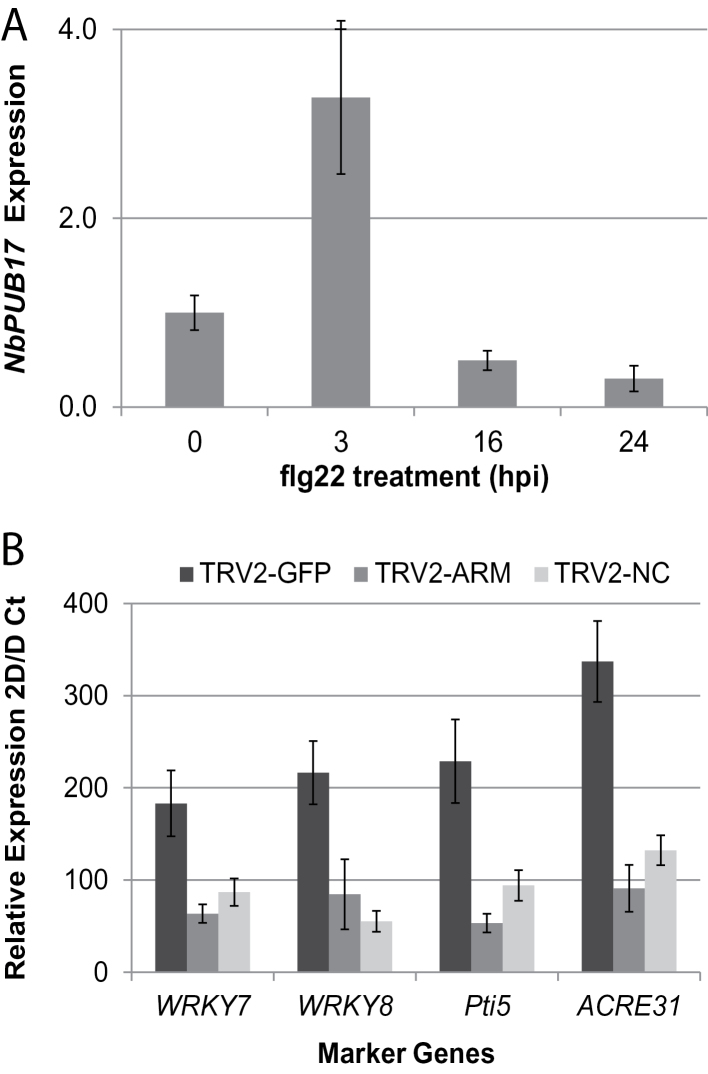
Virus-induced gene silencing of *NbPUB17* in *N*. *benthamiana* attenuates flg22-mediated transcriptional responses. (A) *NbPUB17* transcript accumulation at 3h post-inoculation (hpi) of flg22. (B) Flg22-mediated transcript accumulation for *NbWRKY7, NbWRKY8, NbPti5,* and *NbACRE31* at 3 hpi in TRV2-GFP, TRV2-ARM, and TRV2-NC expressing plants, relative to untreated TRV2-GFP.

It has been reported previously that *AtPUB17* is rapidly up-regulated following treatment with the PAMP flg22 (Navarro *et al.*, 2004). It was therefore investigated whether this was also the case for *NbPUB17* in *N*. *benthamiana*. Following flg22 infiltration into leaves as described previously (McLellan *et al.*, 2013) a discrete peak of *NbPUB17* transcript accumulation was noted specifically at 3h post-inoculation (Fig. 2A). This prompted an investigation into whether PUB17 contributes to the flg22-mediated PTI response, using the early-induced genes *NbWRKY7, NbWRKY8, NbPti5*, and *NbACRE31* (McLellan *et al.*, 2013). Following flg22 treatment of VIGS plants, remarkably, given that PUB17 function has only previously been associated with ETI (Yang *et al.*, 2006), a significantly reduced accumulation of *NbWRKY7, NbWRKY8, NbPti5*, and *NbACRE31* transcripts was observed in TRV::ARM or TRV::NC plants, compared with the TRV::GFP control (Fig. 2B). This indicates that PUB17 contributes to the activation of PTI as well as ETI.

### Silencing of *NbPUB17* in *N*.*benthamiana,* or *NtPUB17* (*NtACRE276*) in tobacco, compromises Cf4/Avr4 cell death, but not that triggered by INF1 or by co-expression of R3a and AVR3a^KI^

**Fig. 3.**
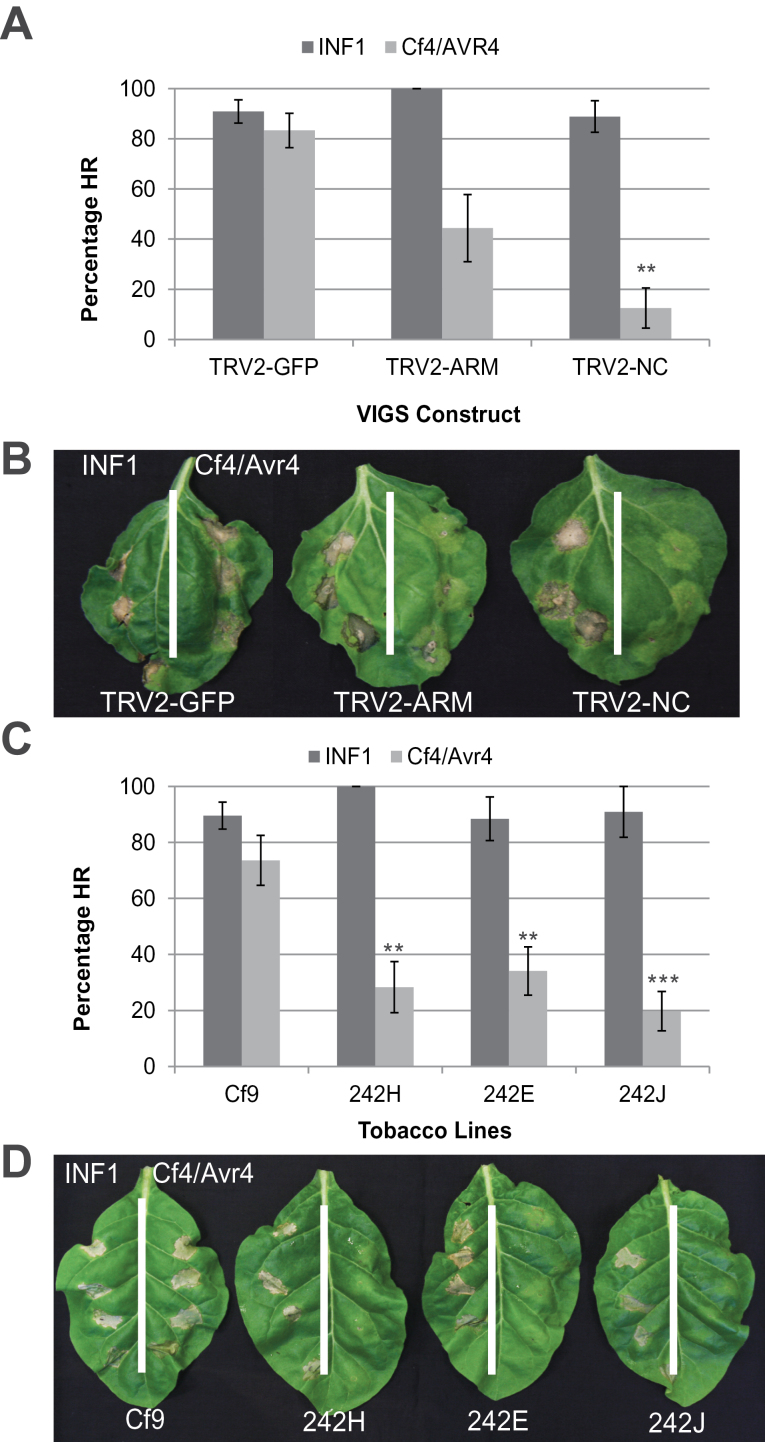
Silencing of *PUB17* in *N*. *benthamiana* or tobacco compromises Cf4/Avr4 cell death but not INF1 cell death. (A) Graph shows the percentage HR elicited by INF1 or Cf4/Avr4 on *N*. *benthamiana* plants silenced for *NbPUB17* using the TRV-ARM and NC constructs, respectively. Statistical analysis was carried out using ANOVA with pairwise comparisons performed with a Holm–Sidak test; **P ≤0.01 (*n*=11), error bars show standard error. (B) Representative leaves silenced for *NbPUB17* and TRV-GFP showing INF1 HR on the left side of the leaf and Cf4/Avr4 HR on the right. (C) Graph shows the percentage HR elicited by INF1 or Cf4/Avr4 on *N*. *tabacum* plants silenced for *NtPUB17* (lines 242H, E, and J) are shown beside the non-silenced control Cf9. Statistical analysis was carried out using ANOVA with pairwise comparisons performed with a Holm–Sidak test; **P ≤0.01, ***P ≤0.001 (*n*=22), error bars show standard error. (D) Representative leaves silenced for *NtPUB17* and the control showing INF1 HR on the left side of the leaf and Cf4/Avr4 HR on the right.

Previous work showed that PUB17 from either tomato or tobacco is a positive regulator of programmed cell death (PCD) triggered by a recognition of the apoplastic effectors Avr4 and AVR9 from *C. fulvum* by the corresponding receptor-like resistance proteins CF4 and CF9 (Yang *et al.*, 2006). This analysis was extended to investigate whether PUB17 is also required for cell death triggered by perception of a PAMP from *P*. *infestans*, the elicitin INF1. In addition to VIGS of *NbPUB17* in *N*. *benthamiana*, the previously published (Yang *et al.*, 2006) tobacco NtPUB17 RNAi lines were also included. As expected, VIGS of *NbPUB17* (Fig. 3A, B), or RNAi of *NtPUB17* (Fig. 3C, D), significantly compromised CF4/AVR4 PCD. However, silencing of *NbPUB17* or *NtPUB17* failed to attenuate INF1 cell death in either plant, indicating that not all cell death events following perception of an elicitor at the cell surface require PUB17 (Fig. 3).

**Fig. 4.**
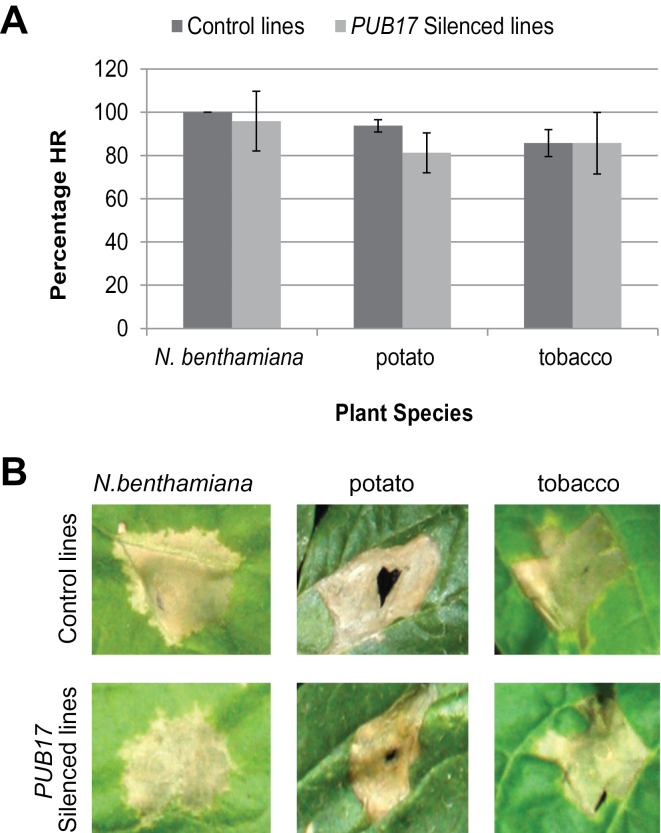
The HR mediated by R3a is not affected by silencing of *PUB17*. (A) Graph shows percentage HR elicited by Agro-infiltration of R3a/Avr3a constructs on *N*. *benthamiana*, potato, and tobacco plants with the control lines (respectively, TRV2-GFP, potato E3, and tobacco Cf9) compared with *PUB17* silenced lines (respectively, TRV2-NC in *N*. *benthamiana,* RNAi-3 in potato, and 242J in tobacco). Statistical analysis carried out by ANOVA indicates no significant differences, error bars show standard error. (B) Representative images showing close up of the HR on the control and *PUB17* silenced lines used for each plant species. All experiments are the combination of at least three biological replicates, each using six or seven plants, and inoculations of at least four leaves from each plant.

To investigate the potential roles of PUB17 orthologues as positive regulators of PCD further, the impact of silencing *NbPUB17, NtPUB17* or *StPUB17* on the HR triggered by the intracellular recognition of AVR3a^KI^ from *P*. *infestans* by potato R3a was studied. This was shown previously to be independent of the E3 ligase, CMPG1, which is another positive regulator of CF4-AVR4-mediated PCD (Bos *et al.*, 2010; Gilroy *et al.*, 2011). It was observed that VIGS of *NbPUB17* in *N*. *benthamiana*, or RNAi of *NtPUB17* in tobacco or *StPUB17* in potato, each failed to attenuate R3a-mediated HR (Fig. 4).

### GFP-StPUB17 and GFP-NtPUB17 strongly accumulate in the plant nucleolus

**Fig. 5.**
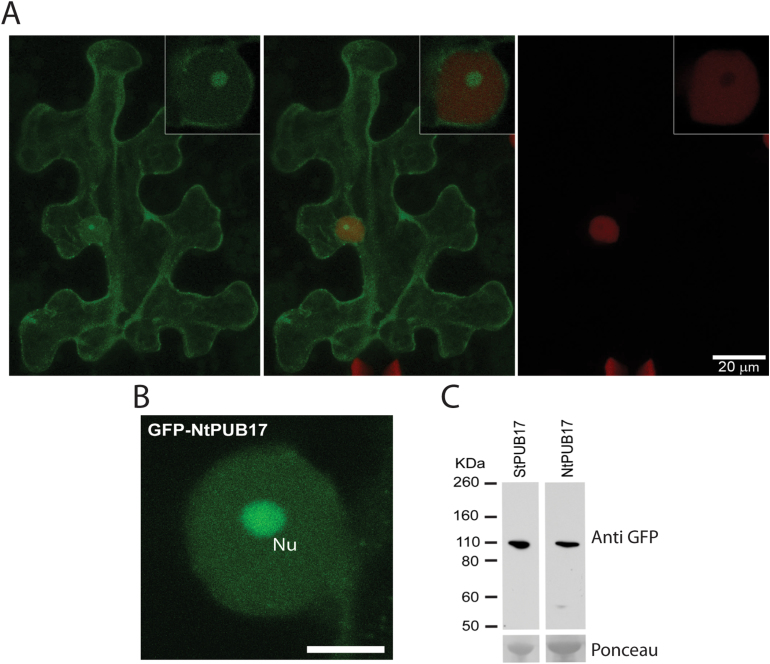
GFP-StPUB17 and GFP-NtPUB17 strongly accumulate in the nucleolus *in planta*. (A) Representative CB157 *N*. *benthamiana* leaves expressing mRFP-H2B examined by confocal microscopy 48h after infiltration of 35S-GFP-StPUB17, indicating green (left), red (right), and merged (centre) channels, with close-up nuclear confocal images inset. (B) Representative close-up nuclear confocal image showing the sub-nuclear localization of GFP-NtPUB17. Nu indicates the nucleolus and the scale bar is 10 μm. (C) Western blots probed with a GFP antibody showing stable protein fusions of potato and tobacco GFP-PUB17 of the expected size. The lower panels show Ponceau staining of the membrane as a loading control.

*StPUB17* was cloned in-frame with an N-terminal *GFP* fusion and transiently expressed in *N*. *benthamiana* to study sub-cellular localization. Strikingly, when expressed in transgenic *N*. *benthamiana* CB157 (expressing mRFP-H2B, which labels the nucleoplasm), it was noted that GFP-StPUB17 protein was observed in the nucleus and especially in the nucleolus (Fig. 5A), with background cytoplasmic fluorescence (see Supplementary Fig. S5 at *JXB* online). GFP-fusion to NtPUB17 demonstrated that it showed similar localizations (Fig. 5B; see Supplementary Fig. S5 at *JXB* online). Immunoblots demonstrated that each GFP-fusion protein was intact (Fig. 5C), indicating that the fluorescence accurately reflects the localization of PUB17 in each case.

### Dominant-negative activity of an StPUB17^V314I,V316I^ mutant is prevented by nuclear exclusion

**Fig. 6.**
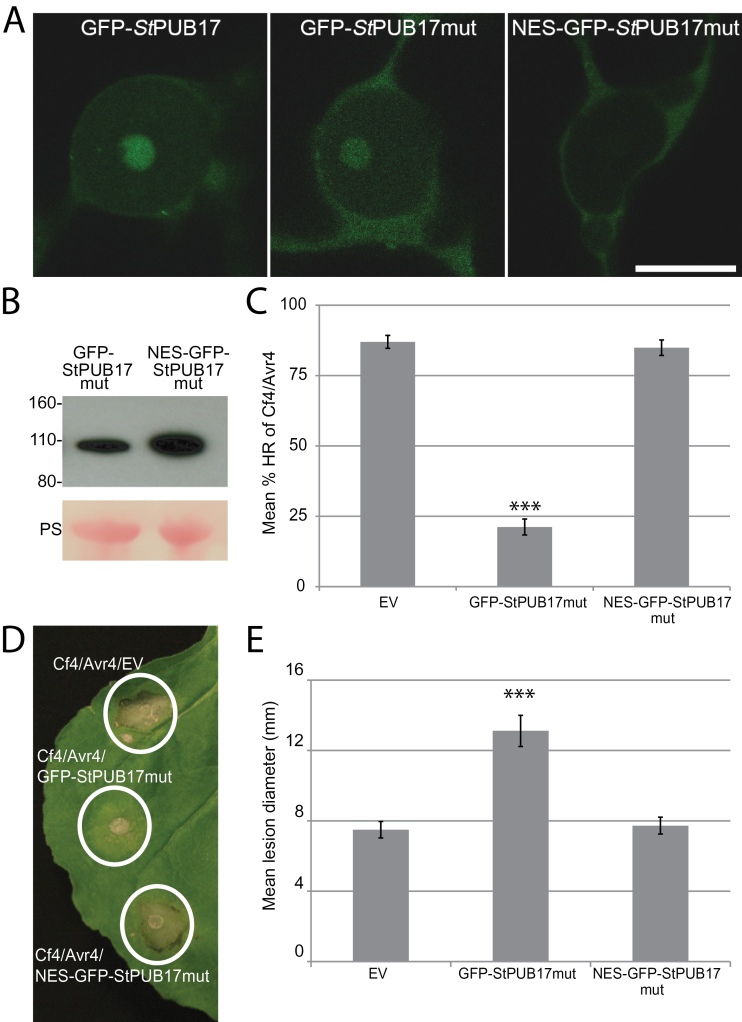
Dominant-negative activity of an StPUB17^V314I,V316I^ mutant (StPUB17mut) is prevented by nuclear exclusion. (A) Representative nuclear images of GFP-STPUB17, GFP-StPUB17mut, and NES-GFP-StPUB17mut. Size marker is 10 μm. (B) Western blots probed with a GFP antibody showing stable protein fusions of GFP-StPUB17mut and NES-GFP-StPUB17mut of the expected size. The lower panels show Ponceau staining (PS) of the membrane as a loading control. (C) Mean % HR of Cf4/Avr4 co-expressed with empty vector (EV), GFP-StPUB17mut, and NES-GFP-StPUB17mut. (D) Images of HR of Cf4/Avr4 co-expressed with empty vector (EV), GFP-StPUB17mut, and NES-GFP-StPUB17mut. (E) Mean lesion diameter of *P*. *infestans* colonization following expression of empty vector (EV), GFP-StPUB17mut, and NES-GFP-StPUB17mut. Statistical analysis was carried out using ANOVA with pairwise comparisons performed with a Holm–Sidak test; ***P ≤0.001 [*n*=92 for (C) and *n*=106 for (E)]; error bars show standard error.

Previously, mutation of AtPUB17 to prevent U-box activity resulted in a mutant form that acted as a dominant-negative to suppress Cf9-mediated cell death (Yang *et al.*, 2006). Therefore, equivalent valine residues in StPUB17 were mutated to isoleucines (V314I, V316I) to create StPUB17^V314I,V316I^ (referred to as StPUB17mut). Similar to WT GFP-StPUB17, GFP-StPUB17mut localized to the nucleus and predominantly to the nucleolus, with a cytoplasmic background (Fig. 6A), and was stable *in planta* (Fig. 6B). Transient co-expression of GFP-StPUB17mut with Cf4 and Avr4 resulted in a significant suppression of cell death (Fig. 6C, D), indicating that our mutant form acts as a dominant-negative, and that this activity is not inhibited by the presence of the N-terminal GFP fusion. Moreover, expression of GFP-StPUB17mut supported significantly enhanced *P*. *infestans* colonization of *N*. *benthamiana* (Fig. 6E). In order to assess whether such dominant-negative activity requires nuclear localization, a nuclear exclusion signal (NES) was fused to the N-terminus of the fused GFP in this construct, creating NES-GFP-StPUB17mut. NES-GFP-StPUB17mut was excluded from the nucleus (Fig. 6A) and was again stable *in planta* (Fig. 6B). However, in contrast to the GFP-StPUB17mut, the NES-GFP-StPUB17mut failed to suppress CF4/Avr4 cell death (Fig. 6C, D) or enhance *P*. *infestans* colonization of *N*. *benthamiana* (Fig. 6E).

## Discussion

Previously, the *Arabidopsis* AtPUB17 protein, and its functional orthologues in tomato and tobacco, SlPUB17 (SlACRE276) and NtPUB17 (NtACRE276), respectively, were shown to be functional ubiquitin E3 ligases that act as positive regulators of the HR triggered by R/AVR recognition events: RPM1/AvrB and RPS4/AvrRPS4 in *Arabidopsis* and Cf4/AVR4 in tomato and tobacco (Yang *et al.*, 2006). In addition, using RNAi, it was shown that StPUB17 in potato is required for basal immunity to *P*. *infestans* (Ni *et al.*, 2010). It was confirmed that silencing of *PUB17* in potato enhances late blight infection and it is shown that this is also the case in the closely-related solanaceous host *N*. *benthamiana*, allowing detailed functional studies to be performed in this plant. The following new observations about PUB17 function have been made: (i) whereas PUB17 is required for CF4/AVR4 cell death, as previously shown (Yang *et al.*, 2006), it is not required for cell death triggered by the *P*. *infestans* PAMP INF1, indicating that PUB17 is not involved in all cell death events triggered following pathogen perception by cell surface receptors; (ii) silencing *PUB17* orthologues in potato, *N*. *benthamiana* or tobacco failed to attenuate R3a/AVR3a^KI^ HR, indicating that PUB17 is not involved in all R/AVR-mediated cell death events; (iii) remarkably, however, it is demonstrated that PUB17 promotes the activation of PTI triggered by the PAMP flg22; and (iv) critically, using a dominant-negative mutant of StPUB17, it is shown that its activity requires nuclear localization. Each of these observations is discussed below.

It has been demonstrated that GFP fusions of StPUB17 and NtPUB17 show a similar nucleo-cytoplasmic location and strongly accumulate in the nucleolus, suggestive of a shared nucleolar function. Interestingly, the E3 ligase CMPG1, which is a target of the *P*. *infestans* effector AVR3a (Bos *et al.*, 2010; Gilroy *et al.*, 2011), also showed nucleolar accumulation when stabilized by the effector, even though AVR3a itself does not localize in the nucleolus. It is therefore likely that both positive regulators of cell death, PUB17 and CMPG1, have nuclear/nucleolar functions, although this has yet to be shown for CMPG1. Nevertheless, whereas CMPG1 is required for both INF1- and CF4-mediated cell death (Gilroy *et al.*, 2011), PUB17 is not required for INF1 cell death, indicating that they may have distinct substrates for ubiquitination, and thus different regulatory roles.

By mutation of the key valine residue required for E3 ligase activity in the U-box, Yang *et al.* (2006) generated a dominant-negative version of AtPUB17 that suppresses Cf-mediated cell death. Therefore, this strategy was utilized to investigate the sub-cellular localization of PUB17 activity. An equivalent dominant- negative StPUB17 form was generated, showing that it also suppressed Cf4-mediated cell death. Importantly, the mutant retained predominantly nuclear/nucleolar localization. By excluding this mutant from the nucleus the dominant-negative activity was lost, indicating that at least some critical StPUB17 substrates for ubiquitination are potentially also located in the nucleus. By contrast, a number of PUB E3 ligases that negatively regulate immunity have been shown to act at the plasma membrane to target and ubiquitinate receptors such as FLS2 (PUB12 and PUB13), or to target components of vesicle trafficking (PUB22) (Lu *et al.*, 2011; Stegmann *et al.*, 2012). Future work will involve identifying the nuclear substrates of PUB17 to determine their fate following ubiquitination accurately.

Previously, AtPUB17 was shown to be required for HR mediated by NB-LRR resistance proteins RPM1 and RPS4, suggesting that it is a positive regulator of ETI (Yang *et al.*, 2006). However, silencing of *PUB17* in potato, tobacco, and *N*. *benthamiana* had no impact on R3a-AVR3a^KI^ mediated HR, suggesting that it is not required for this ETI response, and that this is conserved within the Solanaceae. CMPG1 was also not required for the HR mediated by cytoplasmic NB-LRR proteins such as R3a, R2, and Rx, and seems to be associated with positively regulating cell death triggered by cell-surface recognition events (Gilroy *et al.*, 2011). It is interesting that RPM1 is activated at, and signals from, the plasma membrane (Gao *et al.*, 2011); hence it will be important to explore further any role of PUB17 in signal transduction from the cell surface. It is possible that, although PUB17 substrates probably reside in the nucleus, the protein itself could be activated from outside the nucleus.

*StPUB17* was shown previously to be induced during a compatible potato–*P*. *infestans* interaction as early as 24 hpi (Ni *et al.*, 2010). It is shown here that silencing *StPUB17* in potato by RNAi, and VIGS of *NbPUB17* in *N*. *benthamiana*, each significantly enhance colonization by *P*. *infestans*. Moreover, expression of the dominant-negative GFP-StPUB17mut also enhanced colonization. These results indicate that PUB17 probably contributes to basal immunity to late blight disease during the biotrophic stage of interaction. Silencing results with *PUB17* in potato and *N*. *benthamiana*, in contrast to VIGS results with *CMPG1* which resulted in a reduction in sporulating *P*. *infestans* lesions, further emphasize the different roles that these two E3 ubiquitin ligases play during late blight disease. The *CMPG1* VIGS result was explained by the coincident reduction in *AVR3a* transcript accumulation and the increase in *INF1* expression during the switch from biotrophy to necrotrophy. This implies that the pathogen promotes CMPG1 activity during the necrotrophic phase to trigger host cell death for the benefit of completing its infection cycle (Bos *et al.*, 2010).

How does PUB17 contribute to immunity to late blight? Whilst PUB17 in the Solanaceae plants studied here is required for CF4-mediated HR, it is not involved in the HR triggered by perception of the *P*. *infestans* PAMP, INF1. *P*. *infestans* does not possess an effector related to the *C. fulvum* AVR4 and, to date. no CF-like proteins have been shown to provide resistance to late blight. However, it has recently been shown that the RXLR effector RD2 from *P*. *infestans* enhances colonization of *N*. *benthamiana* by targeting the kinase domain of the host MAP3Kɛ protein, which is required for CF4-mediated cell death, but not for INF1-mediated PCD. Moreover, VIGS of *MAP3Kɛ* led to significantly enhanced *P*. *infestans* colonization (King *et al.*, 2014). This indicates that the signal transduction pathway associated with CF4-mediated immunity is likely also to be triggered by *P*. *infestans* perception, supporting the involvement of PUB17 in basal defence to late blight. Future studies, prompted by observations here and in King *et al.* (2014), will focus on identifying the host receptor(s) and the corresponding pathogen molecules that trigger the CF4/9-associated signal transduction pathway in the *P*. *infestans*–host interaction, and in identifying the substrates of PUB17 to understand better its regulatory role in immunity.

In addition to a potential role, during late blight infection, in promoting a response pathway associated with Cf-mediated cell death, it is shown that PUB17 also acts to promote PTI activated by the PAMP flg22; silencing of *PUB17* significantly attenuated induction of flg22-triggered early marker genes. Whilst *P*. *infestans* does not possess the PAMP flg22, the associated, generic FLS2 signal transduction pathway has recently been shown to be relevant to late blight as it is inhibited, at different stages, by a range of *P*. *infestans* RXLR effectors (Zheng *et al.*, 2014). Thus, the contribution of PUB17 to basal immunity to late blight is likely to include its roles in promoting both Cf- and FLS2-associated signalling pathways. Interestingly, the observation that PUB17 does not contribute to INF1-mediated cell death highlights the differences in signal transduction between cell-surface perception events and, indeed, between different PAMPs. It will be important to identify both the host receptors and *P*. *infestans* elicitors responsible for activating these pathways.

In conclusion, it has been shown that, whereas PUB17 has previously been shown positively to regulate cell death triggered from cell-surface receptors and from intracellular NB-LRR resistance proteins (Yang *et al.*, 2006), it is not involved in all cell death pathways, as exemplified by INF1-mediated and R3a/AVR3a-mediated cell death. However, it has also been shown that it does play a role in early activation of PTI gene expression. Finally, it has been demonstrated that PUB17 acts as a positive regulator of immunity in the host nucleus.

## Supplementary data

Supplementary data are available at *JXB* online.

Supplementary Fig. S1. Alignment of potato, tobacco, and *Arabidopsis* PUB17 amino acid sequences.

Supplementary Fig. S2. StPUB17 functions as an E3 ubiquitin ligase.

Supplementary Fig. S3. Expression analysis of *StPUB17* RNAi lines.

Supplementary Fig. S4. Expression analysis of *NbPUB17* levels in three replicates of virus-induced gene silencing.

Supplementary Fig. S5. Confocal images of potato and tobacco GFP-PUB17 fusion proteins.

Supplementary Table S1. Primers used in this study.

Supplementary Table S2. Genes, constructs, vectors and their use during this study

Supplementary Data
