# Supplementary material for: U-box E3 ubiquitin ligase PUB17 acts in the nucleus to promote specific immune pathways triggered by Phytophthora infestans
Source: J Exp Bot. 2015 Apr 6;66(11):3189–99. doi: 10.1093/jxb/erv128 (PMC4449539; doi:10.1093/jxb/erv128)
Supplement: Supplementary Data [file supp_66_11_3189__index.html]

U-box E3 ubiquitin ligase PUB17 acts in the nucleus to promote specific immune pathways triggered by Phytophthora infestans — U-box E3 ubiquitin ligase PUB17 acts in the nucleus to promote specific immune pathways triggered by Phytophthora infestans — Supplementary Data 

# U-box E3 ubiquitin ligase PUB17 acts in the nucleus to promote specific immune pathways triggered by *Phytophthora infestans*

## Supplementary Data

Data files

**Files in this Data Supplement:**

- Supplementary Data - Supplementary Data
