# Supplementary material for: U-box E3 ubiquitin ligase PUB17 acts in the nucleus to promote specific immune pathways triggered by Phytophthora infestans
Source: J Exp Bot. 2015 Apr 6;66(11):3189–99. doi: 10.1093/jxb/erv128 (PMC4449539; doi:10.1093/jxb/erv128)
Supplement: Supplementary Data [file supp_erv128_jexbot142836_file001.pdf]

Supplementary Information

U-BOX E3 ubiquitin ligase PUB17 acts in the nucleus to promote specific immune pathways triggered by *Phytophthora infestans*

Running title:

E3 ligase PUB17 positively regulates immunity in the nucleus

Authors:

Qin He[1,2,3], Hazel McLellan[2,3], Petra C Boevink[3,5], Ari Sadanandom[4], Conghua Xie[1], Paul R J Birch[2,3,5*], Zhendong Tian[1*]

Address:

[1]Key Laboratory of Horticultural Plant Biology (at HAU), Ministry of Education; National Center for Vegetable Improvement (Central China); Huazhong Agricultural University, Wuhan, Hubei, 430070, China

[2]Division of Plant Sciences, University of Dundee (at JHI); [3]Dundee Effector Consortium; [5]Cell and Molecular Sciences; all at James Hutton Institute (JHI), Errol Road, Invergowrie, Dundee DD2 5DA UK.

[4]Durham Centre for Crop Improvement Technology School of Biological and Biomedical Sciences, Durham University, DH1 3HP, UK.

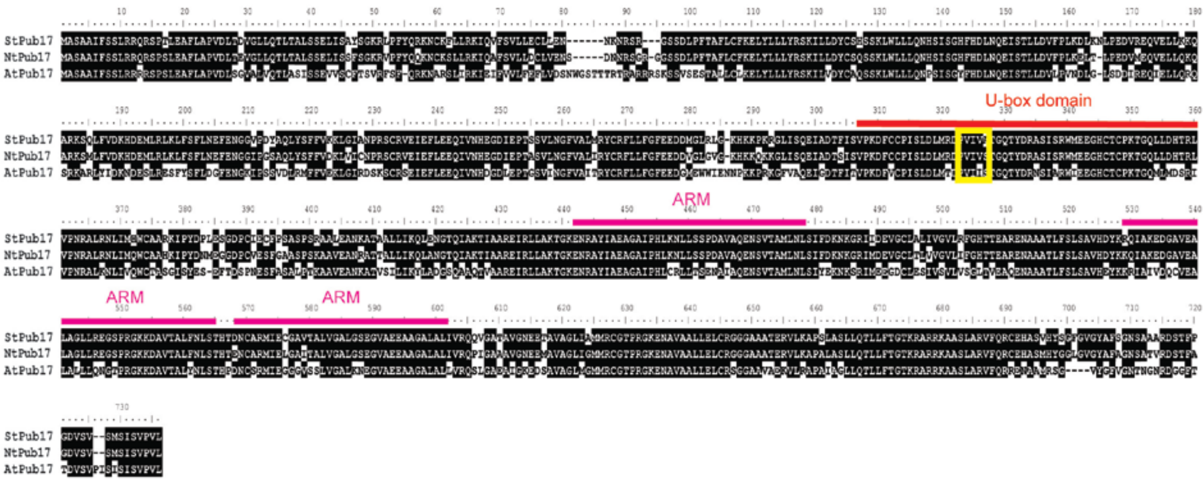

Supplemental Figure S1. Alignment of potato (St), tobacco (Nt) and Arabidopsis (At) PUB17 amino acid sequences. Alignment was carried out using CLUSTALW analysis. Black boxes indicate conserved amino acids. The U-box domain is indicated by a red line and the three ARM domains are indicated with pink lines. A yellow box indicates the VIV sequence that was mutated to III (V314I, V316I) in StPUB17.

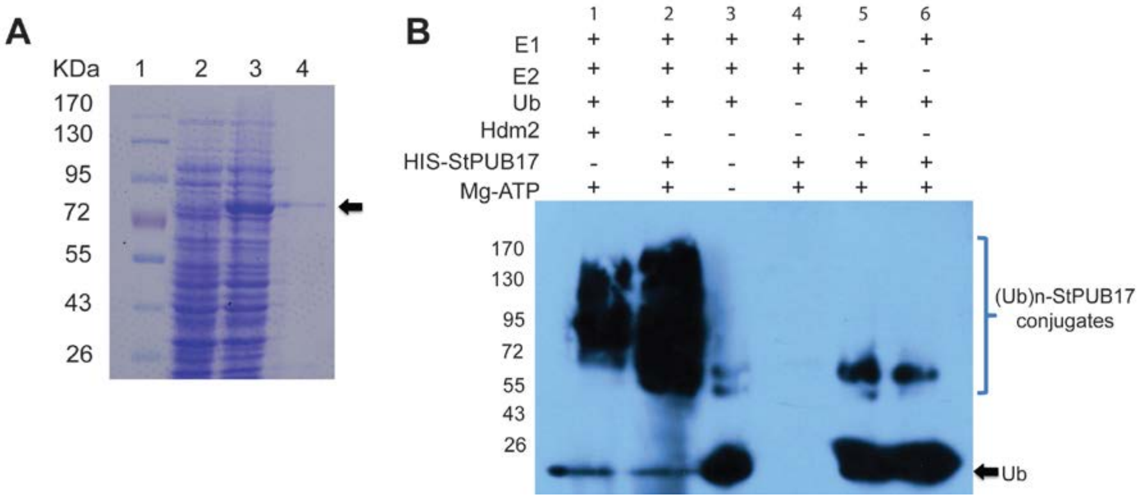

Supplemental Figure S2. StPUB17 Functions as an E3 Ubiquitin Ligase. (a) An SDS PAGE gel stained with coomassie blue, numbered lanes contain 1- Size marker, 2- non-induced cell extract containing StPUB17, 3- induced cell extract containing StPUB17 by 0.2 mM Isopropyl β-D-1-thiogalactopyranoside for 2 h, 4- purified StPUB17. The arrow indicated the expected size of StPUB17. (b) E3 ubiquitin ligase activity assay of StPUB17. Purified HIS-StPUB17 fusion protein was incubated in the presence or absence of E1, E2, ATP, and ubiquitin. The reactions were analyzed with immunoblots using anti-ubiquitin antibody. E3 ubiquitin ligase activity of HIS-StPUB17 was only detected in the presence of E1, E2, ATP, and ubiquitin (lane 2). Hdm2 was used as a positive control (Lane 1); Lanes 3, 4, 5 and 6: Negative control reactions omitting Mg-ATP, Ub, E1 and E2.

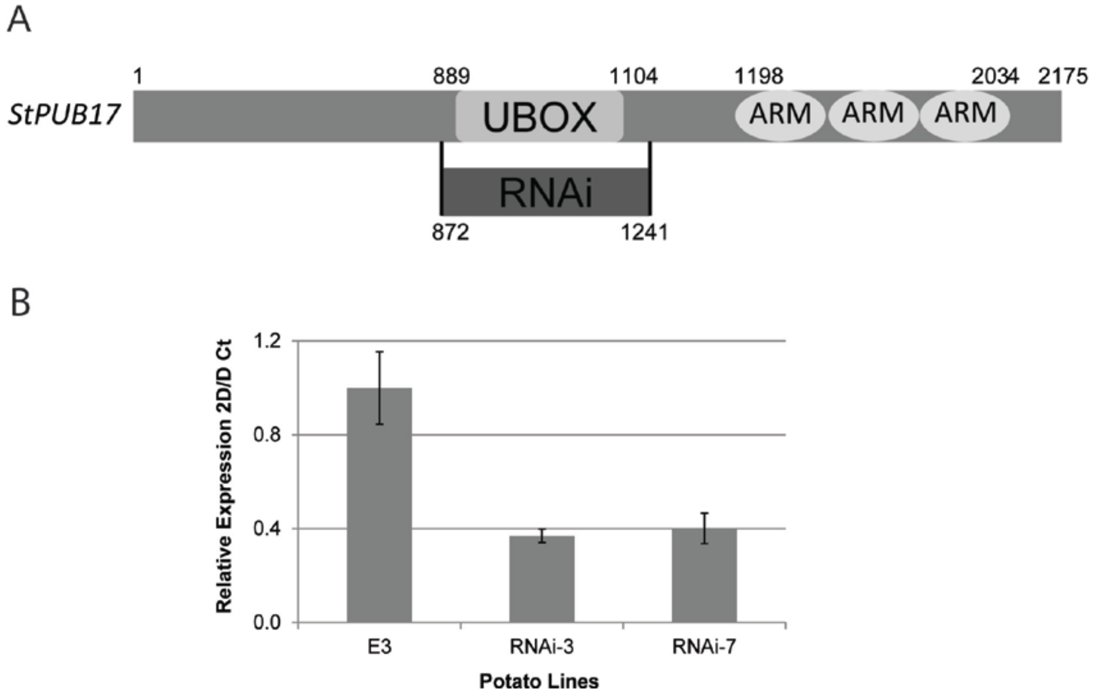

Supplemental Figure S3. Expression analysis of *StPUB17* RNAi lines. (A) Schematic showing the *StPUB17* gene with the base number corresponding to each domain shown along the top. The areas used to generate the RNAi silencing construct and the corresponding basepair numbers are indicated below. (B) Graph shows the expression level of *StPUB17* detected by qRT-PCR in two independent potato lines (RNAi-3, RNAi-7) stably transformed with *StPUB17* RNAi constructs compared to the control (E3). The analysis was performed using the 2Delta/Delta Ct method and the error bars indicate combined standard error.

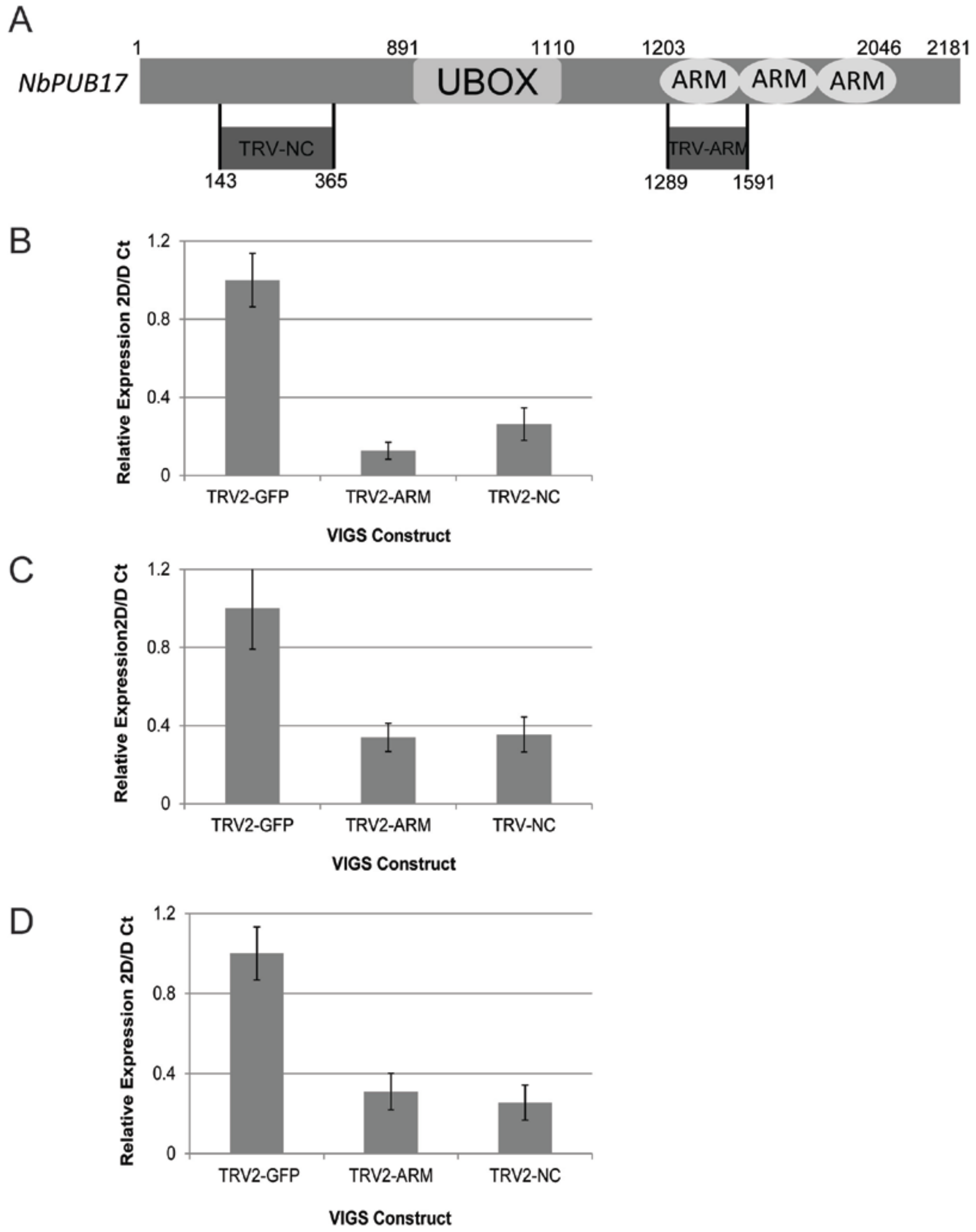

Supplemental Figure S4. Expression analysis of *NbPUB17* levels in three replicates of virus induced gene silencing. (A) Schematic showing the *NbPUB17* gene with the base number corresponding to each domain shown along the top. The areas used to generate the VIGS silencing constructs and the corresponding basepair numbers are indicated below. (B-D) The graphs show the levels of expression of *NbPUB17* detected by qRT-PCR in plants 3 weeks after infiltration with TRV-GFP, TRV-ARM and TRV-NC constructs, the analysis was performed using the 2Delta/Delta Ct method and the error bars indicate combined standard error. Each graph shows an individual biological rep.

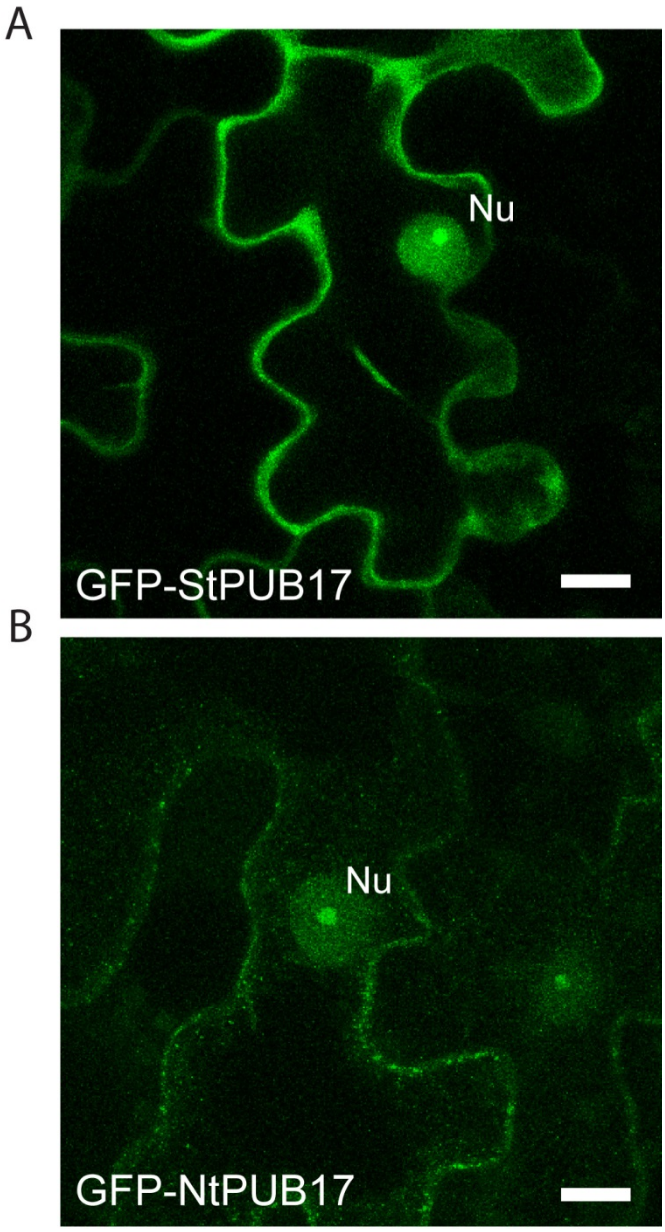

Supplemental Figure S5. Confocal images of potato and tobacco GFP-PUB17 fusion proteins. (A, B) *N. benthamiana* leaves were examined by confocal microscopy 48 h after infiltration of 35s-GFP-PUB17 constructs (as indicated). Representative images for the GFP channel (in green) are shown for each PUB17 fusion as indicated. Nu indicates fluorescence observed in the nucleolus and the scale bar denotes 10μm

Table S1. Primers used in this study.

**Gene**	**Forward Primer**	**Reverse Primer**	**Function**
StPUB17	StPUB17F: AAGAATTCATGGCATCTGCTGCAAT (Introduce EcoRI enzyme site)	StPUB17R: TTGCGGCCGCCTATAATACTGGAACTG (introduce NotI enzyme site)	cloning of StPUB17 to pMD18-T vectors
StPUB17	StPUB17 XbaI F: AATCTAGAATGGCATCTGCTGCAATTT (introduce XbaI enzyme site)	StPUB17 ScaI R: TTGAGCTCCTATAATACTGGAACTGAA (introduce ScaI enzyme site)	potato overexpression transgenic vector
StPUB17	StPUB17-Full-F: AA AAA GCA GGC TTC ACC ATGGCATCTGCTGCAATTTTC	StPUB17-Full-R: A GAA AGC TGG GTC CTATAATACTGGAACTGAAAT	gateway cloning of StPUB17 Full lenghth
StPUB17	StPUB17 RT PCR-F: GGAAGTGAAGGTGTTGCCGA	StPUB17 RT PCR-R: CTACTGCCGTTTCCTCATTGC	qRT_PCR in potato
NbPUB17	NbPUB17 Full-Xba I-F GCTCTAGAATGGCATCAGCTGCAATTTTCTCAT(Xba I)	NbPUB17 Full-Sac I-R:CGAGCTCCTATAACACTGGTACTGAAATGGAC	cloning of NbPUB17 to pMD18-T vectors
NbPUB17	TRV2- NbPUB17-arm F-XbaI: GCTCTAGATAGCAAAAGAAGATGGGGCAGTCG(Xba I)	TRV2- NbPUB17-arm R-SacI: CGAGCTCCACGGCATAATTCAAGCAACGCAG(Sac I)	VIGS of NbPUB17
NbPUB17	TRV2- NbPUB17-NC-Xba I F : GCTCTAGACGGTAAAAGGGTTCCCTTTTATCA (Xba I)	TRV2- NbPUB17-NC-Sac I R: CGAGCTCGATTGGGAGCAATAATCAAGCAAG (Sac I)	VIGS of NbPUB17
NbPUB17	NbPub17AF: CCAGGAGTTGTAGGGTTGAGA	NbPub17AR: ACAAACCCATTGAGCACTGA	qRT_PCR in *N. benthamiana*
NbEf1a	qRT_Nb-ef1aF: TGGACACAGGGACTTCATCA	qRT_Nb-ef1aR: CAAGGGTGAAAGCAAGCAAT	qRT_PCR internal control for *N. benthamiana*
StEf1a	qPCR-*ef1a*-F: ATTGGAAACGGATATGCTCCA	qPCR-ef1a-R:TCCTTACCTGAACGCCTGTCA	qRT_PCR internal control for potato
PiO8-3-3	PiO8-3-3-Fwd: CAAT-TCGCCACCTTCTTCGA	PiO8-3-3-Rev: GCCT-TCCTGCCCTCAAGAAC	monitor the *P. infestans* growth
NbPti5	qRT_Pti5_F: CCTCCAAGTTTGAGCTCGGATAGT	qRT_Pti5_R: CCAAGAAATTCTCCATGCACTCTGTC	qRT_PCR in *N. benthamiana*
NbAcre31	qRT_Acre31_F: AATTCGGCCATCGTGATCTTGGTC	qRT_Acre31_R: GAGAAACTGGGATTGCCTGAAGGA	qRT_PCR in *N. benthamiana*
NbWRKY7	qRT_NbWRKY7_F: CACAAGGGTACAAACAACACAG	qRT_NbWRKY7_R: GGTTGCATTTGGTTCATGTAAG	qRT_PCR in *N. benthamiana*
NbWRKY8	qRT_NbWRKY8_F: AACAATGGTGCCAATAATGC	qRT_NbWRKY8_R: TGCATATCCTGAGAAACCATT	qRT_PCR in *N. benthamiana*
StPUB17mut	SDMPub17IIIF: catacgtctgccctgttgcgataataattggatccctcatcaaatccaac	SDMPub17IIIR: gttggatttgatgagggatccaattattatcgcaacagggcagacgtatg	StPUB17 mutation (314V 316V to 314I 316I)

Table S2. Genes, constructs, vectors and their use during this study

Genes	Gene Constructs	vectors cloned into	experiments
StPUB17	GFP-StPUB17	pB7WGF2	Localisation in *Nicotiana benthamiana*
	GFP-StPUB17mut (GFP-StPUB17^{V314I,V316I})	pB7WGF2	Localisation in *Nicotiana benthamiana* and dominant-negative activity assay
	NES-GFP-StPUB17mut	NES-pB7WGF2 (the NES signal sequence was inserted with annealed oligonucleotides into the unique *Spe*I site at the beginning of the GFP in the pB7WGF2 vector)	Localisation in *Nicotiana benthamiana* and dominant-negative activity assay
	HIS-StPUB17	pET28a	Purification of HIS-StPUB17 fusion protein for E3 ubiquitin ligase activity assay)
	RNAi-3 and -7	pHGRV: The portion spanning the UBOX region of StPUB17 (Supplementary Fig S3) was cloned into pHGRV vector	Silencing StPUB17 in potato by RNAi
NbPUB17	GFP-NbPUB17	pB7WGF2	Localisation in *Nicotiana benthamiana*
	TRV2-ARM (TRV::ARM)	TRV2: Tobacco Rattle Virus (TRV) vector (The portion spanning the ARM repeat-encoding region of NbPUB17 (Supplementary Fig S4) was cloned into TRV2 vector	Silencing NbPUB17 in *Nicotiana benthamiana* by VIGS
	TRV2-NC (TRV::NC)	TRV2: (The portion spanning the NC repeat-encoding region of NbPUB17 (Supplementary Fig S4) was cloned into the TRV2 vector	Silencing *NbPUB17* in *Nicotiana benthamiana* by VIGS
GFP	TRV2-GFP	TRV2: Tobacco Rattle Virus (TRV) vector. The empty GFP was cloned into TRV2 vector	VIGS silencing control
	EV	pB7WGF2	The empty GFP control for transient expression assays *in planta*
